# High-Sensitivity Janus Sensor Enabled by Multilayered Metastructure Based on the Photonic Spin Hall Effect and Its Potential Applications in Bio-Sensing

**DOI:** 10.3390/s24175796

**Published:** 2024-09-06

**Authors:** Xiang Li, Haifeng Zhang

**Affiliations:** College of Electronic and Optical Engineering & College of Flexible Electronics (Future Technology), Nanjing University of Posts and Telecommunications, Nanjing 210023, China; b21021012@njupt.edu.cn

**Keywords:** electromagnetic waves propagation, Janus effect, multilayered metastructure, optical biological detection, photonic spin Hall effect

## Abstract

The refractive index (RI) of biological tissues is a fundamental material parameter that characterizes how light interacts with tissues, making accurate measurement of RI crucial for biomedical diagnostics and environmental monitoring. A Janus sensor (JBS) is designed in this paper, and the photonic spin Hall effect (PSHE) is used to detect subtle changes in RI in biological tissues. The asymmetric arrangement of the dielectric layers breaks spatial parity symmetry, resulting in significantly different PSHE displacements during the forward and backward propagation of electromagnetic waves, thereby realizing the Janus effect. The designed JBS can detect the RI range of 1.3~1.55 RIU when electromagnetic waves are incident along the +*z*-axis, with a sensitivity of 96.29°/refractive index unit (RIU). In the reverse direction, blood glucose concentrations are identified by the JBS, achieving a sensitivity of 18.30°/RIU. Detecting different RI range from forward and backward scales not only overcomes the limitation that single-scale sensors can only detect a single RI range, but also provides new insights and applications for optical biological detection through high-sensitivity, label-free and non-contact detection.

## 1. Introduction

Multilayered metastructures [1] are artificially designed micro-scale optical structures that precisely control the phase [2], frequency [3], and polarization [4] of electromagnetic waves (EMWs) through the careful design of interlayer media, enabling the accurate manipulation of their propagation paths and characteristics [5]. A photonic crystal (PC) [6,7,8,9] is a material with a periodic structure that controls the propagation of EMWs through the photonic band gap [10]. The design principle of a one-dimensional (1D) PC biosensor is based on the fact that when biological molecules enter the structure of the PC or adhere to its surface, they alter the optical properties of the crystal, thereby affecting the EMWs propagation characteristics [11]. By measuring these changes, the detection of biological molecules can be achieved. Due to its low cost, ease of integration, and real-time detection capabilities [12,13,14], this sensor is of significant value in dynamic biological monitoring and chemical analysis [15]. Refractive index (RI) is a crucial optical parameter for biological materials [16]. When biological samples (such as proteins [17], cells [18], or bacterial samples [19]) come into contact with or attach to the sensing surface of the sensor, they cause localized changes in the RI. Small variations in RI can provide valuable analytical information, allowing predictions of biological molecule adsorption, aggregation, or chemical reactions [20]. Aly et al. reported a 1D PC biosensor, whose sensing mechanism relies on the change in the RI of cancer cells to cause the change in the transmission spectrum for detection [21]. Omar A et al. designed a one-dimensional defect PC to locate the resonance peak position of EMWs corresponding to protein solubility, enabling detection functionality [22]. Maria A et al. discovered a correlation between the biological response to bacterial contaminants and the blue shift in photonic response, leading to the development of a plasma PC biosensor [23]. These studies show that by measuring changes in RI, biosensors can detect biological molecules and contaminants with high sensitivity, validating the effectiveness of RI as a key parameter in biosensing.

Optical sensors employ various technologies, such as optical interference [24], optical scattering [25], chemiluminescence [26], and infrared absorption [27]. Among the broad field of modern physics, the photonic spin Hall effect (PSHE) [28] has increasingly become a focus of optical research due to its superior performance in detecting minute changes. The PSHE occurs when linearly polarized light passes through a material with optical inhomogeneities, leading to spatial separation of photons with different spin states and resulting in photon spin Hall displacement [29]. This effect arises because of the strong spin-orbit coupling between the spin and the orbital angular momentum of the beam [30]. Specifically, PSHE manifests as the separation of spin and right circularly polarized components of light during reflection or refraction at interfaces between media with different RI [31]. The physical mechanism of PSHE is analogous to the Electronic Spin Hall Effect (ESHE), where the RI arrangement of the dielectric layer parallels the potential gradient in ESHE [32]. The high sensitivity of PSHE to minor changes in system parameters makes it a valuable tool for characterizing weak RI and nanostructure parameter variations [33]. Typically, the spin splitting displacement in ESHE is at the nanoscale and challenging to observe [34]. However, the introduction of weak measurement techniques has amplified the observable PSHE phenomenon, enhancing its detection ability [35].

Sensitivity is one of the main parameters of optical sensors [36]. Sensitivity can be divided into absolute sensitivity and relative sensitivity [37]. Relative sensitivity takes into account the working range of wavelength [37]. For general RI optical sensors, absolute sensitivity is usually expressed as the change in sensor output signal caused by a unit change in physical quantity [36]. Its absolute sensitivity can be defined as the change in RI of the analysis layer corresponding to the translation of spectral frequency and wavelength. High-sensitivity RI sensing has important applications in human blood type identification [38]. Janus, the Roman god of creation, has two distinct faces [39]. Researchers have achieved asymmetric propagation of electromagnetic waves by using the asymmetric arrangement of dielectric layers [40]. This phenomenon is called the Janus property and represents the two-sided physicality [40]. In sensor design, by utilizing the Janus effect, RI measurements of different organisms can be achieved under forward and backward incident EMWs, and sensing of different physical quantities can be performed in positive and negative scales to realize multi-scale sensor functions [40].

Aly [21], Omar A [22], and Maria A et al. [23] achieved single-scale biological sensing. However, the design of multi-scale sensors that have different sensitivities and detection ranges for the measured physical quantities in the forward and backward scales, and the detection of different biological quantities in the forward and backward scales, still has huge development potential. In this paper, a Janus sensor (JBS) composed of layered metastructures with different media was proposed based on the PSHE. At EMW frequency of 71 GHz, the JBS allows for RI detection in the range of 1.3 refractive index unit (RIU) to 1.55 RIU with a sensitivity of 96.29°/RIU. When EMWs are propagated in the negative direction with a frequency of 71 GHz, the JBS can distinguish blood glucose concentration, with a sensitivity of 18.30°/RIU, achieving biosensing functionality. Specific JBS detection metrics are shown in Table 1. It should be noted that the focus of this work is on theoretical validation, as practical experiments were beyond the scope due to limitations in equipment and funding. However, feasible experimental schemes still deserve discussion. In terms of device manufacturing, the layered structure can be manufactured by the etching method, and the relevant manufacturing technology is already very mature [41]. The application of weak measurement technology makes it possible to build experimental equipment and can effectively detect weak PSHE displacements [42]. Thus, this innovative biosensor design provides new insights for the development of multi-scale devices.

## 2. Structure Design and Simulation

**Figure 1:**
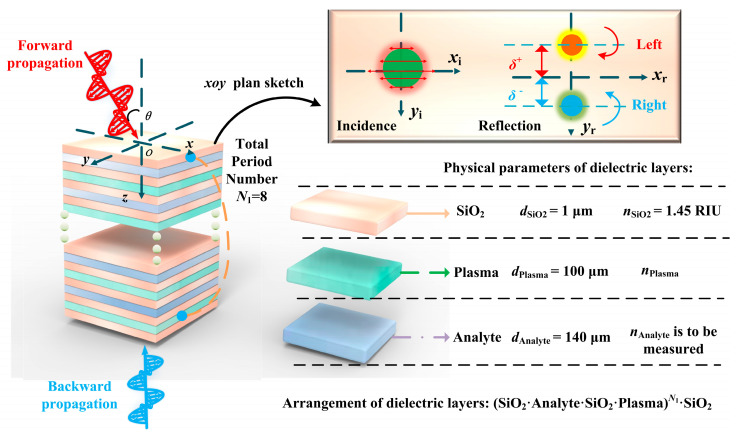
A schematic representation of the JBS is described as (SiO_2_·Analyte·SiO_2_·Plasma)*^N^*^1^·SiO_2_, where the number of cycles *N*_1_ = 8. The upper right corner is the schematic of the photon spin Hall effect, and the lower right corner is the physical parameters of the dielectric layer.

The asymmetric arrangement of the layered metastructure breaks the spatial symmetry, achieving Janus functionality. Figure 1 illustrates the arrangement sequence of the layers in the JBS. The layers of SiO_2_, Plasma, and Analyte are arranged along the +*z*-axis in the order (SiO_2_·Analyte·SiO_2_·Plasma)*^N1^*·SiO_2_, where *N*_1_ = 8 represents the number of periods in the dielectric layers. The entire JBS is placed in the air at an ambient temperature of 300 K to suit typical sensor application scenarios. EMWs incident on the JBS from different directions exhibit different electromagnetic spectra. Forward and backward incidences are indicated by red and blue arrows, respectively, within the *xoz*-plane. The angle *θ* denotes the incident angle, which is the angle between the incident EMWs and the *z*-axis. The upper right corner of Figure 1 shows the process of photonic spin Hall displacement. The glowing spheres on the left and right sides represent the situations when the EMWs are incident and reflected, respectively. *x*_i_ and *y*_i_ denote the reference coordinate system for the incident EMWs, while *x*_r_ and *y*_r_ denote the reference coordinate system for the reflected EMWs. When a linearly polarized light with an RI gradient reflects on the surface of the structure, it splits into left-handed and right-handed circularly polarized EMWs, with beam displacements denoted as *δ*^+^ and *δ*^−^, respectively. The beam displacements for vertical and horizontal reflected EMWs are denoted as *δ*_V_ and *δ*_H_. *d*_SiO2,_ *d*_Plasma_, and *d*_Analyte_ represent the thicknesses of each dielectric layer, with dimensions of 1 μm, 100 μm, and 140 μm, respectively. The RI of SiO_2_ is *n*_SiO2_ = 1.45 RIU [43], the RI of Analyte is *n*_Analyte_ (to be specified in subsequent sections), and the RI of Plasma is represented as follows [44]:

(1) $$\varepsilon_{\mathrm{Plasma}}=1-\frac{\omega_{p}^{2}}{\omega (\omega +j\gamma )},n_{\mathrm{Plasma}}=\sqrt{\varepsilon_{\mathrm{Plasma}}}$$

Here, *ω_p_* = (e^2^ × *n*_e_/*ε*_0_/m)^1/2^ represents the plasma frequency, where *n*_e_ = 1 × 10^20^ m^−3^ is the plasma density [44]. *ε*_0_ = 8.8542 × 10^−12^ F/m denotes the vacuum permittivity. e = 1.6 × 10^−19^ C and m = 9.1 × 10^−31^ Kg [44] are the electron charge and mass, respectively. *ω* = 2π*f* represents the angular frequency of the incident wave, where *f* = 71 GHz is the frequency of the incident EMWs. *γ* = 0.001*ω_p_* is the plasma collision frequency [44].

The transfer matrix method can be used to calculate the energy propagation between layers in the JBS, with the transfer matrices for each layer represented as [28]:

(2) $$MT_{i}=\left(\begin{array}{cc} \cos(k_{iz}d_{i}) & -\frac{i}{\eta_{i}}\sin(k_{iz}d_{i})\\ -i\eta_{i}\sin(k_{iz}d_{i}) & \cos(k_{iz}d_{i}) \end{array}\right),$$

Here, *i* in ***MT**_i_* can represent SiO_2_, plasma, and analyte, symbolizing the transmission matrices of different media. *k_iz_* = *ω/cn_i_* sin *θ_i_* [28] is the component of the wave vector along the *z*-axis, where *c* is the speed of EMWs in a vacuum. The *s*-wave is a vertically polarized wave with its electric field direction perpendicular to the incident plane, while the *p*-wave is a horizontally polarized wave with its electric field direction parallel to the incident plane. *η_i_* is the optical conductivity, where *η_i_* = (*ε*_0_/*μ*_0_)^1/2^*n*_i_ cos *θ_i_* for *s*-waves and *η_i_* = (*ε*_0_/*μ*_0_)^1/2^*n_i_*/cos *θ_i_* for *p*-waves [28]. *μ*_0_ is the magnetic permeability of the vacuum. The energy transfer for the structure (SiO_2_·Analyte·SiO_2_·Plasma)*^N^*^1^·SiO_2_ is represented as [29]:

(3) $$MT_{\mathrm{total}}={\left(MT_{SiO_{2}}\cdot MT_{\mathrm{Analyte}}\cdot MT_{\mathrm{Plasma}}\cdot MT_{SiO_{2}}\right)}^{8}\cdot MT_{SiO_{2}}=\left(\begin{array}{cc} m_{a} & m_{b}\\ m_{c} & m_{d} \end{array}\right).$$

The reflection (*r*) and transmission (*t*) coefficients can be expressed as [29]:

(4) $$r=\frac{\left(m_{a}+m_{b}\eta_{0})\eta_{0}-(m_{c}+m_{d}\eta_{0}\right)}{\left(m_{a}+m_{b}\eta_{0})\eta_{0}+(m_{c}+m_{d}\eta_{0}\right)},$$

(5) $$t=\frac{2\eta_{0}}{\left(m_{a}+m_{b}\eta_{0})\eta_{0}+(m_{c}+m_{d}\eta_{0}\right)}.$$

*R* = |*r*|^2^ and *T* = |*t*|^2^ represent the reflectivity and transmission, respectively [28].

The displacement shown in Figure 1 is also due to the PSHE phenomenon on a dielectric interface produced by a narrow Gaussian beam at an angle of *θ*. It can be represented as follows [32]:

(6) $${\tilde{E}}_{i\pm }=(e_{ix}+ioe_{iy})\frac{\omega_{0}}{\sqrt{2\pi }}\exp\left[-\frac{\omega_{0}^{2}\left(k_{ix}^{2}+k_{iy}^{2}\right)}{4}\right]$$

Here, *ω*_0_ and *o* represent the beam waist and polarization operator. *k_ix_*, *k_iy_* are the components of the wave vector in the direction of the *x*-axis and the *y*-axis, respectively. Left-handed (*o* = 1) and right-handed (*o* = −1) circularly polarized light is represented as *o*, respectively. When the waist radius of the Gaussian beam is large enough and the transmission distance is relatively short, the Gaussian beam can be approximated as a plane wave. Then, the connection between the incident field and the reflected field was constructed [32].

(7) $$\left[\begin{array}{c} {\tilde{E}}_{r}^{H}\\ {\tilde{E}}_{r}^{V} \end{array}\right]=\left[\begin{array}{cc} r_{p} & \frac{k_{ry}\cot\theta_{i}(r_{p}+r_{s})}{k_{0}}\\ -\frac{k_{ry}\cot\theta_{i}(r_{p}+r_{s})}{k_{0}} & r_{s} \end{array}\right]\left[\begin{array}{c} {\tilde{E}}_{i}^{H}\\ {\tilde{E}}_{i}^{V} \end{array}\right].$$

Here, *k*_0_ represents the wave number in free space, and in the *y_r_* direction, *k_ry_* is the wave vector component of the reflected beam. The Fresnel reflection coefficients for *s*-wave and *p*-wave are denoted as *r_s_* and *r_p_*, respectively. By combining Equations (6) and (7), the expression of the reflection angular spectrum can be derived [28].

(8) $${\tilde{E}}_{r}^{H}=\frac{r_{p}}{\sqrt{2}}[\exp(+ik_{ry}\delta_{r}^{H}){\tilde{E}}_{r+}+\exp(-ik_{ry}\delta_{r}^{H}){\tilde{E}}_{r-}],$$

(9) $${\tilde{E}}_{r}^{V}=\frac{ir_{s}}{\sqrt{2}}[-\exp(+ik_{ry}\delta_{r}^{V}){\tilde{E}}_{r+}+\exp(-ik_{ry}\delta_{r}^{V}){\tilde{E}}_{r-}].$$

Here, *δ_r_^H^* = (1 + *r_s_*/*r_p_*) cos *θ_i_*/*k*_0_ and *δ_r_^V^* = (1 + *r_p_*/*r_s_*) cos *θ_i_*/*k*_0_. For reflected EMWs, the transverse displacements of the PSHE can be obtained [32].

(10) $$\delta_{H}^{\pm }=\mp \frac{\lambda }{2\pi }[1+\frac{\left|r_{s}\right|}{\left|r_{p}\right|}\cos(\varphi_{s}-\varphi_{p})]\cot\theta_{i},$$

(11) $$\delta_{V}^{\pm }=\mp \frac{\lambda }{2\pi }[1+\frac{\left|r_{p}\right|}{\left|r_{s}\right|}\cos(\varphi_{p}-\varphi_{s})]\cot\theta_{i}.$$

This paper focuses on the changes of *δ*_v_^−^ to realize the sensing function.

## 3. Results and Discussion

**Figure 2:**
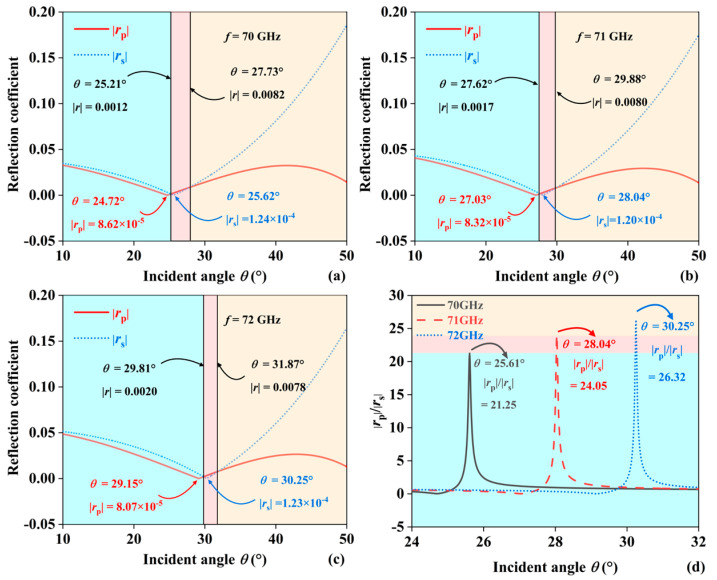
The Fresnel reflection coefficient of (**a**) *f* = 70 GHz, (**b**) *f* = 71 GHz, (**c**) *f* = 72 GHz, *P*-wave and *S*-wave at different EMWs incident frequencies. (**d**) Fresnel reflection coefficient ratio of *P*-waves and *S*-waves.

To determine the reflection coefficients of EMWs at different incident frequencies, the optimal peak of *δ*_v_^−^ was selected to discuss the reflection coefficients for different incident frequencies *f*. Frequencies of 70, 71, and 72 GHz were chosen with 1 GHz intervals. Since the effective RI of the JBS responds differently to EMWs at different frequencies, the propagation of EMWs is affected [32]. This is reflected in the Fresnel reflection coefficient in Figure 2. Figure 2a–c illustrates the relationship between the Fresnel reflection curves of |*r*_s_|and |*r*_p_| within an incident angle range of 10° to 50° for different frequencies. |*r*_p_| is represented by a red solid line, and |rs| is represented by a blue dashed line. Figure 2a shows the reflection coefficients at an incident EMW frequency of 70 GHz. There are two intersection points in the curves of |*r*_s_|and |*r*_p_|, at 25.21° (|*r*| = 0.0012) and 27.73° (|*r*| = 0.0082). In the ranges of 10° to 25.21° and 27.73° to 50°, |*r*_s_| is greater than |*r*_p_|, while in the range of 25.21° to 27.73°, |*r*_s_| is less than |*r*_p_|. The minimum value of |*r*_p_| is 8.62 × 10^−5^, and the minimum value of |*r*_s_| is 1.24 × 10^−4^. Figure 2b shows the reflection coefficients at an incident EMWs frequency of 71 GHz. At 27.62° (|*r*| = 0.0017) and 29.88° (|*r*| = 0.0080), |*r*_s_| = |*r*_p_|. In the ranges of 10° to 27.62° and 29.88° to 50°, |*r*_s_| is greater than |*r*_p_|, while in the other range, |*r*_s_| is less than |*r*_p_|. The minimum value of |*r*_p_| is 8.32 × 10^−5^ at an incident angle of 27.03°, and the minimum value of |*r*_s_| is 1.2 × 10^−4^ at an incident angle of 28.04°. Figure 2c shows the reflection coefficients at an incident EMWs frequency of 72 GHz. In the ranges of 10° to 29.81° and 31.87° to 50°, |*r*_s_| is greater than |*r*_p_|, while in the remaining range, |*r*_s_| is less than |*r*_p_|. The minimum value of |*r*_p_| is 8.07 × 10^−5^ at an incident angle of 29.15°, and the minimum value of |*r*_s_| is 1.23 × 10^−4^ at an incident angle of 30.25°. According to equation (11), the PSHE displacement *δ*_v_^−^ mainly depends on the |*r*_p_|, |*r*_s_| ratio. The larger |*r*_p_| and the smaller |*r*_s_|, the greater the PSHE displacement. Figure 2d shows the values of |*r*_p_|/|*r*_s_| at 70, 71, and 72 GHz. At 70 GHz, with *θ* = 25.61°, the maximum value of |*r*_p_|/|*r*_s_| is 21.25; at 71 GHz, with *θ* = 28.04°, the maximum value is 24.05; and at 72 GHz, with *θ* = 30.25°, the maximum value is 26.32. The value of |*r*_p_|/|*r*_s_| is an important inducer of the SPHE displacement.

**Figure 3:**
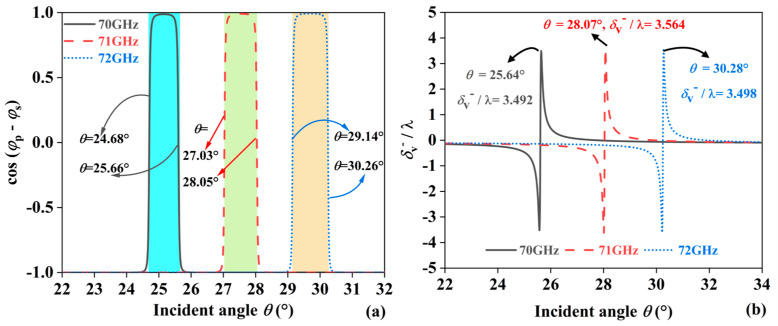
(**a**) cos (*φ*_p_ − *φ*_s_) and (**b**) δ_V_^−^/λ with different *θ* at 70, 71 and 72 GHz.

It should be noted that another reason for the enhanced lateral displacement of the JBS is the variation in the phase difference cos(*φ_p_ − φ_s_*) between *p* − polarized and *s* − polarized light. Figure 3a shows cos(*φ_p_ − φ_s_*) for different frequencies, revealing that as the frequency increases from 70 GHz to 72 GHz, the range where cos(*φ_p_ − φ_s_*) = 1 gradually shifts to larger angles. When the incident EMWs are at 70 GHz, cos(*φ_p_ − φ_s_*) = 1 in the angular range of 24.68° to 25.66°. For 71 GHz, cos(*φ_p_ − φ_s_*) = 1 in the angular range of 27.03° to 28.05°. For 72 GHz, cos(*φ_p_ − φ_s_*) = 1 in the angular range of 29.14° to 30.26°. The displacement size, *δ*_v_^−^/λ, is explained by this variation in Figure 3b. At incident frequencies of 70, 71, and 72 GHz, the maximum values of *δ*_v_^−^/λ are 3.492, 3.564, and 3.498, respectively, at angles of 25.64°, 28.07°, and 30.28°.

**Figure 4:**
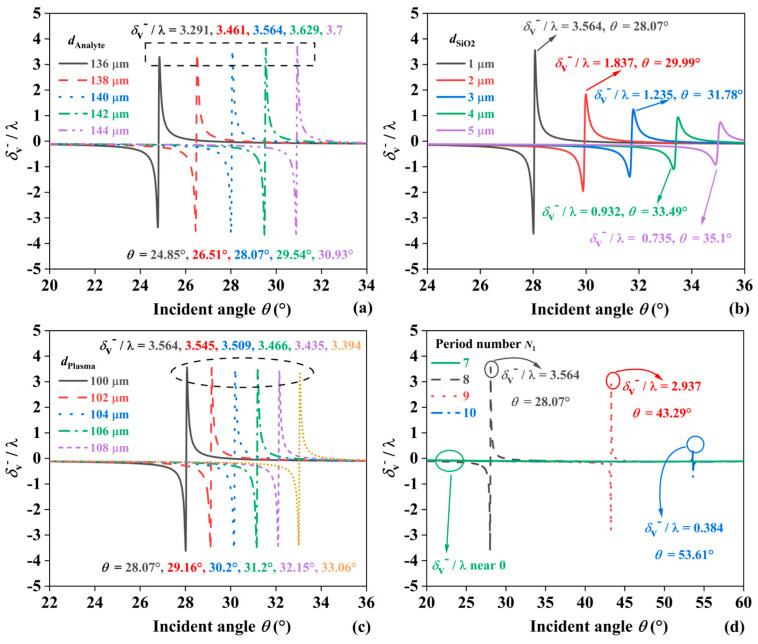
The effect of (**a**) *d*_Analyte_, (**b**) *d*_SiO2_ and (**c**) *d*_Plasma_ changes on δ_V_^−^/λ, and (**d**) the effect of the number of media cycles *N*_1_ on δ_V_^−^/λ.

The PSHE phenomenon is excited in a multilayer structure composed of SiO_2_, Plasma, and Analyte, so the size parameters of these three materials and the number of dielectric periods also affect PSHE. In Figure 4a, *d*_Analyte_ represents the thickness parameter of the Analyte layer. Significant differences in the PSHE effect are observed. When *d*_Analyte_ is 136 μm, 138 μm, 140 μm, 142 μm, and 144 μm, the maximum PSHE displacements are 3.291λ, 3.461λ, 3.564λ, 3.629λ, and 3.7λ, respectively, with corresponding resonance angles of 28.07°, 29.99°, 31.78°, 33.49°, and 35.1°. This indicates that as the thickness of the Analyte increases, the displacement gradually decreases. Figure 4b illustrates the modulation of PSHE displacements with varying SiO_2_ thickness. When the thickness of SiO_2_ rises from 1 μm to 5 μm in increments of 1 μm, the maximum value of *δ*_v_^−^/λ gradually reduces from 3.564 to 0.735, and the resonance angle increases. Specifically, the maximum value of *δ*_v_^−^/λ occurs, corresponding to a resonance angle of 28.07°, whereas the minimum value of *δ*_v_^−^/λ appears, corresponding to a resonance angle of 35.1°. This indicates that variations in SiO_2_ thickness significantly impact both the PSHE displacement and the associated resonance angle. Figure 4c shows the effect of varying Analyte layer thickness on *δ*_v_^−^/λ, with thickness ranging from 100 μm to 108 μm in 2 μm increments. The maximum displacements are 3.564λ, 3.545λ, 3.509λ, 3.466λ, 3.435λ, and 3.394λ, occurring at 28.07°, 29.16°, 30.2°, 31.2°, 32.15°, and 33.06°, respectively. In Figure 4d, when the number of periods *N*_1_ is 7, 8, 9, and 10, the displacement is nearly zero at *N*_1_ = 7, with the maximum displacement occurring at *N*_1_ = 8. For *N*_1_ = 9 and *N*_1_ = 10, the maximum PSHE displacements are 2.937λ and 0.384λ, with corresponding resonance angles of 43.29° and 53.61°.

**Figure 5:**
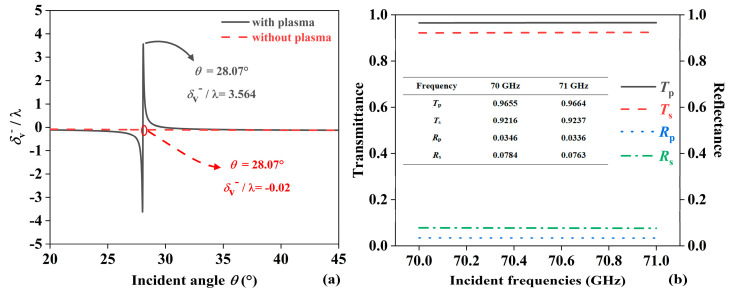
(**a**) The enhancement effect of the plasma. (**b**) The transmission and reflectance of the JBS.

Figure 5a shows the influence of plasma on PSHE displacements within the angular range of 20° to 45°. When the plasma layer is present, the maximum value of *δ*_v_^−^/λ is 3.564, and the corresponding angle is 28.07°. When the plasma is absent, at an incident angle of 28.07°, the value of *δ*_v_^−^/λ is −0.02. Thus, it can be seen that the presence of plasma guides the propagation of EMWs. By reasonably introducing plasma, *δ*_v_^−^/λ can be effectively increased. Figure 5b shows the transmission and reflectance when plasma is present. For the p-polarization and s-polarization, their transmission and reflectivity are expressed as *T*_p_, *T*_s_, *R*_p_ and *R*_s_, respectively. When the incident angle is 28.07°, in the range of 70 ~ 71 GHz, the transmittance of p-polarization and s-polarization are greater than 0.9, and the transmittance of p-polarization is greater than that of s-polarization. At this time, the transmittance of p-polarization at 70 GHz and 71 GHz are 0.9655 and 0.9664, respectively. And at this time, the reflectivity levels are all lower than 0.1. The reflectivity of p-polarization is less than that of s-polarization. The reflectivity under s-polarization at 70 GHz and 71 GHz are 0.0784 and 0.0763, respectively. From the above analysis, it is clear that structural parameters and material design affect the light path distribution of EMWs in the JBS, thereby causing shifts in displacement and angles. This provides a theoretical basis for arbitrary control of PSHE and increases the potential for practical applications.

**Figure 6:**
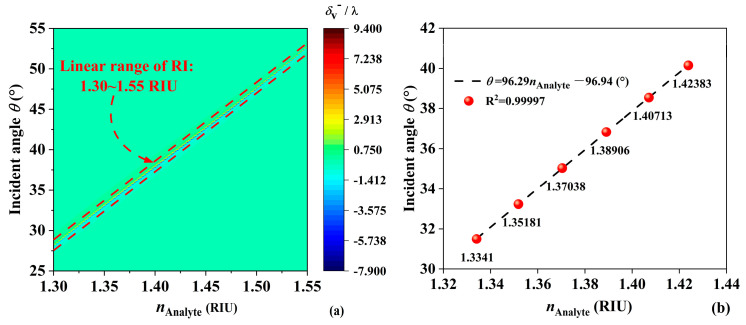
(**a**) The δ_V_^−^/λ of *n*_Analyte_ for forward propagation. (**b**) Fit of angle of incidence and *n*_Analyte_ during forward propagation.

When EMWs are incident at a frequency of 71 GHz, different frequency characteristics are observed for forward and backward propagation. Figure 6a shows the relationship between the RI of the analyte and the PSHE displacement when the wave is incident at various angles. As the *n*_Analyte_ increases from 1.3 RIU to 1.55 RIU, the incident angle also increases. Using the maximum *δ*_v_^−^/λ as the criterion, a linear relationship is observed between the incident angle and the *n*_Analyte_, with a detection range for RI from 1.3 RIU to 1.55 RIU. At *n*_Analyte_ = 1.3341 RIU, the angle is 31.5°, with *δ*_v_^−^/λ of 4.854. As the *n*_Analyte_ increases, *δ*_v_^−^/λ gradually increases, reaching 8.022 at *n*_Analyte_ = 1.42383 RIU, with a corresponding angle of 40.15°. The specific detection parameters are shown in Table 2. Figure 6b describes the linear range of the incident angle with respect to *n*_Analyte_, with the fitting equation *θ* = 96.29*n*_Analyte_ − 96.94°, and R^2^ = 0.99997. The value of R^2^ approaching 1 indicates a good-fitting result [28]. In addition, the detection of protein concentration in aqueous solutions is beneficial for the quantitative study of protein levels and plays a crucial role in disease diagnosis. In fact, the refractive index of proteins is also related to the working frequency band of the sensor, ambient temperature, and acidity and alkalinity. However, when these limitations are ignored and the RI of proteins is assumed to be constant, the refractive index data in Table 2 correspond to protein aqueous solutions with concentrations of 0 to 50 nmol/L (concentration interval is 10 nmol/L) [17,22], and the designed JBS has potential value in protein detection.

**Figure 7:**
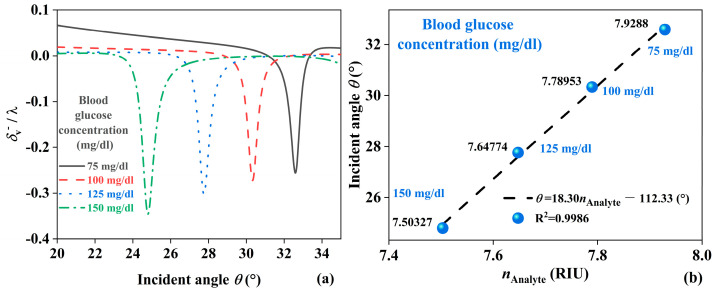
(**a**) Backward EMW propagation to detect *n*_Analyte_. (**b**) Fitting of the incidence angle and the blood glucose concentration.

Figure 7a shows the detection of changes in *n*_Analyte_ when EMWs are incident from the reverse direction. It displays how *δ*_v_^−^/λ varies as the relative permittivity of the analyte changes from 62.8658 to 56.299, with incident angles ranging from 20° to 35°. Table 3 shows the detection of JBS for blood glucose concentration [45,46]. The minimum values of *δ*_v_^−^/λ occur at angles of 32.59°, 30.33°, 27.76°, and 24.8°. As the relative permittivity of the analyte increases, the incident angle also increases. Using the minimum *δ*_v_^−^/λ as the criterion, a linear relationship is observed between the incident angle and *n*_Analyte_. Since different blood glucose concentrations correspond to different dielectric constants, by measuring the refractive index under different blood glucose concentrations, the minimum *δ*_v_^−^/λ will show a significant difference. In the blood glucose concentration range of 75 mg/dL to 150 mg/dL, Figure 7b describes the linear range of the incident angle with respect to blood glucose concentration, with the fitting equation *θ* = 18.30 *n*_Analyte_ − 112.33°, and R² = 0.9986. This indicates a 99.86% confidence level in the linear relationship between the incident angle and *n*_Analyte_, validating the feasibility of the biosensor for blood glucose concentration detection.

Table 4 shows the previous work. By comparison, it is found that the JBS designed in this paper has a significant advantage in detection sensitivity after adopting the PSHE method, and the multi-scale measurement achieved simultaneously provides a new idea for the design of biosensors.

## 4. Conclusions

A JBS that enables the transmission of different ranges of RI from the forward and backward propagation of EMWs was designed in this paper. After discussing the incident EMW frequency, thickness parameters of the dielectric layers, and the number of periods, it was found that increasing the thickness of different dielectric layers generally causes the maximum PSHE displacement to shift towards larger angles. Additionally, appropriate choices of incident frequency and dielectric period numbers also influence the peak value of the maximum displacement and its corresponding angle, allowing for dynamic coordination and tuning of the PSHE displacement angle. The forward propagation of EMWs can detect an RI range of 1.3~1.55 RIU, while backward propagation can detect blood glucose concentration, with maximum detection sensitivities exceeding 95°/RIU, achieving high-sensitivity biosensing.

## Figures and Tables

**Table 1:** Multi-scale detection performance of the JBS.

Scale	Biosensing	JBS Performance of Detection	
Forward	None	Range (RIU)	1.3~1.55
		Sensitivity (°/RIU)	96.29
Backward	Blood glucose concentration	Range (RIU)	7.50327~7.9288
		Sensitivity (°/RIU)	18.30

**Table 2:** RI detection in the forward directions of JBS [17,22].

RI (RIU)	Positioning Angle (°)	*δ*_v_^−^/λ
1.3341	31.5	4.854
1.35181	33.24	5.483
1.37038	35.03	6.261
1.38906	36.83	6.794
1.40713	38.55	7.747
1.42383	40.15	8.022

**Table 3:** RI detection in the backward directions of JBS [45,46].

Blood Glucose Concentration (mg/dL)	Relative Permitivity	Positioning Angle (°)	*δ*_v_^−^/λ
75	62.8658	32.59	−0.2562
100	60.6768	30.33	−0.2723
125	58.4879	27.76	−0.3025
150	56.299	24.8	−0.3457

**Table 4:** Comparison of the published reports with the performance aspects presented by JBS.

Refs.	Multi-Scale	Principle	Detection	Sensitivity
[47]	×	Bloch surface waves	RI: 1.33 ~1.34 RIU	25.1°/RIU
[48]	×	Lossy mode resonance	RI: 1.33~1.45 RIU	61.922°/RIU
[49]	×	Tamm state	RI: 1.333~1.33862 RIU	21.89°/RIU
[50]	×	Surface plasmon resonance	RI	53.96°/RIU
[51]	√	Bloch Surface Wave	RI	0.1046°/RIU
			Temperature	−0.0027°/°C
[52]	×	PSHE	RI	81°/RIU
JBS	√	PSHE	RI: 1.3~1.55 RIU	96.29°/RIU
			Blood glucose concentration	18.30°/RIU

## Data Availability

Dataset available on request from the authors.
